# COVID-19 infections among HCWs exposed to a patient with a delayed diagnosis of COVID-19

**DOI:** 10.1017/ice.2020.256

**Published:** 2020-05-27

**Authors:** Meghan A. Baker, Chanu Rhee, Karen Fiumara, Carin Bennett-Rizzo, Robert Tucker, Sarah A. Williams, Paige Wickner, Jennifer Beloff, Casey McGrath, Alexa Poulton, Michael Klompas

**Affiliations:** 1 Division of Infectious Diseases, Brigham and Women’s Hospital, Boston, Massachusetts; 2 Department of Population Medicine, Harvard Medical School/Harvard Pilgrim Health Care Institute, Boston, Massachusetts; 3 Department of Quality and Safety, Brigham and Women’s Hospital, Boston, Massachusetts; 4 Department of Occupational Health, Brigham and Women’s Hospital, Boston, Massachusetts; 5 Division of Allergy and Clinical Immunology, Brigham and Women’s Hospital, Boston, Massachusetts

## Abstract

We report on COVID-19 risk among HCWs exposed to a patient diagnosed with COVID-19 on day 13 of hospitalization. There were 44 HCWs exposed to the patient before contact and droplet precautions were implemented: of these, 2 of 44 (5%) developed COVID-19 potentially attributable to the exposure.

Healthcare workers (HCWs) are very concerned about their risk of contracting COVID-19 from patients. Researchers in China reported 3,387 infections among HCWs (4.4% of all cases), with 23 attributable deaths.^1^ The Italian National Institute of Health reported that 17,000 HCWs have been infected (~10% of Italy’s cases),^2^ and the US Centers for Disease Control and Prevention (CDC) reported that >9,200 HCWs were diagnosed with COVID-19 in the United States between February 12 and April 9, 2020.^3^ Many HCWs remain concerned that the personal protective equipment (PPE) recommended by the CDC may not be adequate and that SARS-CoV-2 may be transmissible by the airborne route.^4^ Few data, however, have formally quantified the risk of infection in the healthcare setting. Herein, we report on infection risk among a group of HCWs who provided care for a hospitalized patient with COVID-19 without PPE due to delayed diagnosis of COVID-19.

## Methods

A 79-year-old man with a history of type 2 diabetes, hypertension, and a right knee replacement was transferred to our acute-care hospital from a rehabilitation center, complaining of abdominal pain and shortness of breath. He was found to have cholecystitis and underwent a percutaneous cholecystostomy. His shortness of breath and abdominal pain improved while in the intensive care unit although he had an intermittent cough. He was transferred to an intermediate care unit on hospital day 5. On hospital day 13, he developed sudden, acute respiratory failure. Contact and droplet precautions (including eye protection) were instituted. The patient required urgent intubation and was transferred back to intensive care where he was tested for SARS-CoV-2 by nasopharyngeal swab. Reverse transcription polymerase chain reaction (RT-PCR) confirmed a diagnosis of COVID-19.

In retrospect, we felt that the patient likely had undiagnosed COVID-19 since the time of admission. We therefore identified all HCWs who were exposed to the patient during the first 13 days of his hospitalization while he was in a single room on standard precautions. We defined an exposure as ≥10 cumulative minutes of face-to-face contact within 2 meters (6 feet). During this period, the patient was not wearing a mask. The HCWs began wearing surgical masks 7 days into the patient’s hospitalization, based on a hospital policy for universal masking of all providers that was implemented at that time.

We estimated time spent with the patient based on employee’s self-reports, nurse manager assessment, medical record review, and the role of each HCW. All employees were contacted for further details and to assess for symptoms. An employee was considered symptomatic if they had any of the following signs or symptoms: fever or feeling feverish, shortness of breath, cough, sore throat, muscle aches, nasal congestion, or new loss of smell. All staff who met the exposure definition, regardless of symptoms, were offered nasopharyngeal PCR testing for SARS-CoV-2.

## Results

Overall, 44 HCWs met the exposure definition. The median cumulative time spent with the patient was 45 minutes (range, 10–720 minutes). Of the 44 exposed workers, 8 of 44 developed symptoms, of whom 3 tested positive for COVID-19. The remaining 36 exposed workers did not develop symptoms. Of these, 29 of 36 were tested, and all results were negative.

Of the 3 HCWs who tested positive for COVID-19, one was also exposed to a household member with confirmed COVID-19. The household member shared a room with the infected employee and developed symptoms prior to the employee; hence, we attributed this employee’s infection to their household member. The other 2 employees had no known COVID-19 contacts outside of the workplace. Our net impression, then, was that 2 of 43 exposed HCWs developed COVID-19 (4.7%).

The 2 HCWs who were exposed to the patient and subsequently developed symptoms had 60 and 70 minutes of cumulative exposure to the patient respectively within the 14 days preceding their infections. One spent ~60 minutes cumulatively helping to bathe, reposition, and reorient the patient. Most of this time was unmasked and included frequent face to face contact. The other HCW examined the patient daily, wearing a mask for ~50% of the examinations. That provider, while wearing a mask, also placed and secured a nasogastric tube that caused the patient to cough and gag.

## Discussion

Despite substantial exposure to a patient with COVID-19 without adequate PPE, <5% of exposed healthcare personnel tested positive for SARS-CoV-2. The 2 healthcare personnel who did become infected had extended face-to-face contact with the patient during routine care, examinations, and a procedure that induced coughing.

A unique feature of our report is that we tested both symptomatic and asymptomatic exposed healthcare personnel. Most reports have only tested exposed HCWs who developed symptoms.^5^ Asymptomatic and presymptomatic infection has been well documented and identified as a source of transmission in some cases.^6^ We therefore deemed it important to characterize the rate of asymptomatic infection among exposed HCWs. Reassuringly, all 29 of the exposed asymptomatic HCWs who were tested had negative results.

The 2 HCWs who became infected with COVID-19 had considerable contact with the patient, including examining or repositioning the patient without any PPE as well as performing a procedure that induced a cough or gag with only a surgical mask. However, despite spending a median 45 minutes of contact time with the patient, most HCWs did not develop infection despite not wearing the PPE recommended by the CDC. This finding is consistent with other studies of SARS-CoV-2 transmission risk among exposed HCWs.^5,7^ The CDC reported, for example, that only 3 of 121 HCWs who cared for an undiagnosed patient ultimately diagnosed with COVID-19 in California were diagnosed with COVID-19.^5^ Risk factors for transmission in that analysis were aerosol-generating procedures and prolonged contact (>2 hours). Notably, asymptomatic exposed workers were not tested as part of this evaluation.

The strengths of this report include complete symptom follow-up on all healthcare providers who were exposed and testing results from a large set of exposed but asymptomatic HCWs. We were also able to obtain rich details on household, community, and healthcare-related exposures among HCWs who tested positive for COVID-19 to determine the most likely source of infection. Conversely, limitations of our report include the potential for recall error and bias, the fact that the report is limited to a single exposure, and a change in our institution’s mask policy partway through the exposure period, which may have led to more mask use than we have appreciated.

This report adds to the growing literature quantifying the risk of COVID-19 in the healthcare setting, documenting that <5% of HCWs caring for a patient with a delayed diagnosis of COVID-19 developed infections themselves. This case also underscores the need for robust screening of all hospitalized patients for symptoms consistent with COVID-19, both on presentation and daily throughout the hospital stay, coupled with a low threshold for testing. Our experience with this instance of delayed diagnosis due to atypical presentation also suggests the potential value of testing of all patients for SARS-CoV-2 if they require hospital admission.
